# Stress–Charge Nonlinear Physical Description and Tensor Symmetries for Piezoelectric Materials

**DOI:** 10.3390/ma16093432

**Published:** 2023-04-28

**Authors:** A. F. Jaramillo-Alvarado, A. Torres Jacome, P. Rosales-Quintero, H. Vazquez-Leal, G. Diaz-Arango, J. Huerta-Chua, J. Martínez-Castillo

**Affiliations:** 1 Electronics Department, Instituto Nacional de Astrofísica, Óptica y Electrónica (INAOE), Luis Enrique Erro # 1, Tonantzintla, Puebla 72840, Mexico; 2 Electronic Instrumentation Faculty, Universidad Veracruzana, Cto. Gonzalo Aguirre Beltran S/N, Xalapa, Veracruz 91000, Mexico; 3 Tecnologico Nacional de Mexico, Instituto Tecnologico Superior de Poza Rica, Luis Donaldo Colosio Murrieta S/N, Arroyo del Maiz, Poza Rica, Veracruz 93230, Mexico; 4 Research Center in Micro and Nanotechnology, Universidad Veracruzana, Boca del Río, Veracruz 94294, Mexico

**Keywords:** nonlinear piezoelectric devices, stress–charge formulation, tensor symmetry structure, high-order tensors, nonlinear piezoelectric applications, tunable piezoelectric devices

## Abstract

Nonlinear piezoelectric materials are raised as a great replacement for devices that require low power consumption, high sensitivity, and accurate transduction, fitting with the demanding requirements of new technologies such as the Fifth-Generation of telecommunications (5G), the Internet of Things (IoT), and modern radio frequency (RF) applications. In this work, the state equations that correctly predict the nonlinear piezoelectric phenomena observed experimentally are presented. Furthermore, we developed a fast methodology to implement the state equations in the main FEM simulation software, allowing an easy design and characterization of this type of device, as the symmetry structures for high-order tensors are shown and explained. The operation regime of each high-order tensor is discussed and connected with the main nonlinear phenomena reported in the literature. Finally, to demonstrate our theoretical deductions, we used the experimental measurements, which presented the nonlinear effects, which were reproduced through simulations, obtaining maximum percent errors for the effective elasticity constants, relative effective permittivity, and resonance frequencies of 0.79%, 2.9%, and 0.3%, respectively, giving a proof of the potential of the nonlinear state equations presented for the unifying of all nonlinear phenomena observed in the piezoelectric devices.

## 1. Introduction

Piezoelectric materials have been used in several application fields because their performance and set of physical properties meet the requirements in a wide scope of applications. Since the discovery of the piezoelectric effect by the Curie brothers in the 1880s, these types of materials were mainly used in transduction applications, until the 1970s, when their implementation in radio frequency (RF) applications was developed [1], and currently, the semiconductor manufacturing process allows their use in applications where the transduction between mechanical and electric fields is mandatory at the micro-scale.

Amorphous piezoelectric materials are used in applications where miniaturization is not required, and for this reason, currently, crystalline piezoelectric materials dominate the market and industry, mainly with microelectromechanical system (MEMS) devices due to the reproducibility of their physical and system properties. Consequently, the research on these materials is focused on crystalline composites that have high chemical resistance, high breakdown voltages, and high rigidity for RF applications.

Since its discovery, several fabrication techniques have been developed to obtain piezoelectric materials, where the chemical-based techniques have been of interest due to the requirements of thin-film technologies [2]. Deposition techniques such as metal–oxide chemical vapor deposition (MOCVD) [3] and chemical solution deposition (CSD) [4] are current research topics. Furthermore, there are CMOS-compatible deposition techniques, since these processes have low fabrication temperatures, such as sputtering-based techniques, which can obtain high levels of crystallinity [5,6], being an ideal fabrication process to apply the nonlinear phenomena of piezoelectric materials in a new scope of applications [7,8,9,10].

Currently, the main applications of piezoelectric materials are embedded in the MEMS scope, because they use the accurate transduction capability to implement them in several types of applications such as micro and nano-resonators [11,12], energy harvesters [13], accelerometers [14], wearable devices [15], micro- and nano-actuators [16], and sensors for gasses [17] and electrostatic charge [18]. In general, the applications cited share demanding requirements such as low power consumption, high sensitivity, accurate transduction, great chemical resistance, and good enough electrical and mechanical properties, where all of these conditions are met by piezoelectric materials. The modeling of the mentioned devices using the linear description of traditional state equations [19] gives acceptable errors by its predictions; nevertheless, under relatively high electric fields (>$10^{6}$ V/m) and deformation, the physical behavior of materials is not predicted correctly [7,20], and the need for a complete first-principles physical description of the nonlinear phenomena for piezoelectric materials emerges as a mandatory tool for new designs in demanding applications of the industry, such as the Fifth-Generation of telecommunications (5G) and the Internet of Things (IoT).

There are applications that use the nonlinear properties with the same targets as the linear applications exposed above such as actuators [21,22], energy harvesters [23], sensors [24], memories [25], and tunable devices [7,26,27]. In all of these works, the physical and electrical behavior of the system is explained through mathematical models [10,28,29,30,31] or first-principles deductions (a specific thermodynamic formulation) [32,33,34,35], where the models are only valid for a specific geometry disposition or layer stack, while the physical formulations are general, but very difficult to solve analytically. The case of the hysteresis nonlinear effect is a special topic since its behavior has remnant fields after time; its formulation in deformation–charge form and micro-mechanical modeling was exposed in [36,37] respectively. In the models cited, the core concept used is the algebraic or complex expansion of the material parameters, resulting in adjustments of the macroscopic magnitudes of the physicalsystem, e.g., resonance frequency, effective material constants, lumped elements of equivalent circuits, and quality factor, among others. All of these results produce an imbalance of the state equations, being the core problem of models for nonlinear piezoelectric applications since the introduction of adjustment parameters in the material constants reproduces the macroscopic behavior of the phenomena; nevertheless, the physical behavior of the effect is not described by the equations. In contrast, the first-principles formulations are based on the balance of the microscopic states of the physicalsystem, resulting in the prediction of the physical behavior of the macroscopic states, being a complete physical description of the nonlinear effects where the state equations remain balanced; consequently, the solver’s calculus is more difficult and time consuming. Due to this, to include this type of device within the integrated circuit (IC) industry, mathematical tools are needed that allow fast design and manufacturing processes, such as the finite-element-method (FEM)-based design accompanied by compatibility with the main IC fabrication processes, such as CMOS, PD-SOI, FD-SOI, and FinFET. In summary, a thermodynamic formulation with easy implementation in leading FEM simulation software (e.g., COMSOL Multiphysics and COVENTOR) is mandatory for the inclusion of nonlinear piezoelectric devices within the semiconductor industry, and that set of demanding characteristics for the mathematical and physics tools are contained in the formulation presented in this work.

To simulate the nonlinearities and physical behavior of the piezoelectric materials, it is necessary to know the nonlinear state equations with an easy methodology to include them in the FEM solver’ calculus; consequently, the symmetry structure of high-order tensors must be given as well. Despite this, the methodologies found in the literature to implement nonlinear behaviors in leading FEM simulation software are complicated to carry out, and at the same time, the symmetry structures cannot be found (only some components for a few types of materials [32]). For these reasons, the nonlinear applications reviewed cannot be explained by a unified set of equations with known tensor structures, making the industrial adoption of these types of applications more difficult despite their advantages.

Taking into account the above discussion, in this work, we present a complete physical description of the nonlinear behavior of piezoelectric materials, obtained through the deduction from first-principles of the nonlinear state Equations (until third-order phenomena), the transformation laws required, and the symmetry structures of the tensors, for each of the thirty-two point groups of symmetry (all types of crystalline materials). Furthermore, a methodology with an easy way to implement the state equations and high-order tensors components in the main FEM simulation software is presented, allowing designing and manufacturing devices that can be used in the 5G, IoT, and RF application scopes. Finally, this work gives the MEMS scientific community all the mathematical and physics tools needed to research new types of applications and optimizations for nonlinear piezoelectric devices.

## 2. Stress–Charge Nonlinear Formulation

A suitable thermodynamic representation for including the nonlinear effect within FEM simulators is the stress–charge formulation due to the characteristics of direct solvers, since the physical behavior of the electrical permittivity and elasticity constants are well-known parameters of crystalline materials; in the literature can be also found references to perform the energy and dissipation calculus [38].

The following deductions are focused on crystalline materials. The theoretical development starts from first-principles using the Voigt form for mechanical tensors, the Einstein sum convention, and the recommended notation for point groups of symmetry by the International Union of Crystallography (IUCr) [39]. The entropy and the temperature contributions were neglected due to the solid phase of materials, the low power dissipation (around 10 mW/mm${}^{2}$), and the nonlinear perturbative operation regime of the devices. In the next sections, we discuss the experimental limits that govern the theoretical development presented.

From the eight possible formulations [19], we used the thermodynamic potential of the electric Gibbs function [40], the total differential of which is defined for the piezoelectric effect as

(1) $$\begin{array}{c} dG_{2}=-D_{k}dE_{k}+T_{\lambda }dS_{\lambda }, \end{array}$$

where $D_{k}$, $E_{k}$, $T_{\lambda }$, and $S_{\lambda }$ are the electric displacement vector, electric field, stress field, and deformation field, respectively. Therefore, considering the properties of the total differential of a multivariable function, the total differentials for dependent variables are

(2) $$\begin{array}{c} dT_{\lambda }=C_{\lambda \mu }dS_{\mu }-e_{k\lambda }dE_{k},\\ dD_{i}=\epsilon_{ij}dE_{j}+e_{i\mu }^{T}dS_{\mu }, \end{array}$$

where $e_{k\lambda }^{T}$ are the piezoelectric coefficients, $C_{\lambda \mu }$ the elastic constants, and $\epsilon_{ij}$ the electrical permittivity.

To deduce the nonlinear formulation, we expanded the tensor coefficients in Equation (2) through a Taylor series centered at zero and took into account that the dependent variables are a function of $S_{\lambda }$ and $E_{k}$, then the elastic constants are

(3) $$\begin{array}{c} \begin{array}{c} C_{\lambda \mu }=\frac{\partial T_{\lambda }}{\partial S_{\mu }}={\left.\frac{\partial T_{\lambda }}{\partial S_{\mu }}\right|}_{0}+{\left.\frac{\partial^{2}T_{\lambda }}{\partial S_{\mu }\partial S_{\nu }}\right|}_{0}S_{\nu }+{\left.\frac{\partial^{2}T_{\lambda }}{\partial S_{\mu }\partial E_{k}}\right|}_{0}E_{k}, \end{array} \end{array}$$

where the high-order derivatives were measured at constant deformation and the electric field equals zero. Through an analogous procedure, we can obtain all coefficient tensors of Equation (2) as a function of $S_{\lambda }$ and $E_{k}$.

Since Equation (1) is a total differential, we have

(4) $$\begin{array}{c} \begin{array}{c} \frac{\partial G_{2}}{\partial S_{\lambda }}=T_{\lambda },\;\;\mathrm{and}\;\;\frac{\partial G_{2}}{\partial E_{k}}=-D_{k}, \end{array} \end{array}$$

then, considering that $G_{2}$ is a physical magnitude, it is continuous, has an exact differential, and has derivatives up to third-order, and knowing the mixed derivatives equivalence, we obtain

(5) $$\begin{array}{c} \begin{array}{c} -\frac{\partial^{3}G_{2}}{\partial E_{k}\partial S_{\lambda }\partial S_{\mu }}=\frac{\partial^{3}G_{2}}{\partial S_{\lambda }\partial E_{k}\partial S_{\mu }}=\frac{\partial^{3}G_{2}}{\partial S_{\lambda }\partial S_{\mu }\partial E_{k}} \end{array} \end{array}$$

Applying Equations (4) and (5), we can define $g_{\lambda \mu k}$ as

(6) $$\begin{array}{c} \begin{array}{c} \frac{\partial^{2}T_{\lambda }}{\partial E_{k}\partial S_{\mu }}=\frac{\partial^{2}T_{\lambda }}{\partial S_{\mu }\partial E_{k}}=-\frac{\partial^{2}D_{k}}{\partial S_{\lambda }\partial S_{\mu }}=g_{\lambda \mu k} \end{array} \end{array}$$

Considering the other tensors’ coefficients in Equation (2) and applying the same procedure for Equations (3) to (6), we define the remaining high-order tensors as

(7) $$\begin{array}{c} \begin{array}{c} \frac{\partial^{2}T_{\lambda }}{\partial E_{j}\partial E_{k}}=-\frac{\partial^{2}D_{k}}{\partial S_{\lambda }\partial E_{j}}=-\frac{\partial^{2}D_{k}}{\partial E_{j}\partial S_{\lambda }}=q_{kj\lambda }, \end{array} \end{array}$$

and

(8) $$\begin{array}{c} \begin{array}{c} \frac{\partial^{2}T_{\lambda }}{\partial S_{\mu }\partial S_{\nu }}=t_{\lambda \mu \nu }\;,\;\;\frac{\partial^{2}D_{i}}{\partial E_{j}\partial E_{k}}=r_{ijk}. \end{array} \end{array}$$

Subsequently, by replacing Equations (3), (6), (7), and (8) in (2), we obtain

(9) $$\begin{array}{c} dT_{\lambda }=C_{\lambda \mu }dS_{\mu }-e_{k\lambda }dE_{k}+g_{\lambda \mu k}d\left(S_{\mu }E_{k}\right)+q_{\lambda jk}E_{j}dE_{k}+t_{\lambda \mu \nu }S_{\nu }dS_{\mu }\;,\\ dD_{i}=\epsilon_{ij}dE_{j}+e_{\mu i}^{T}dS_{\mu }-q_{ij\mu }d\left(E_{j}S_{\mu }\right)-g_{\lambda \mu i}S_{\lambda }dS_{\mu }+r_{ijk}E_{k}dE_{j} \end{array}$$

After integrating Equation (9), we finally obtain the nonlinear state equations for the piezoelectric effect considering effects up to third-order

(10) $$\begin{array}{c} T_{\lambda }=C_{\lambda \mu }S_{\mu }-e_{k\lambda }E_{k}+\frac{t_{\lambda \mu \nu }}{2}S_{\mu }S_{\nu }+g_{\lambda \mu k}S_{\mu }E_{k}+\frac{q_{jk\lambda }}{2}E_{j}E_{k},\\ D_{i}=\epsilon_{ij}E_{j}+e_{i\mu }^{T}S_{\mu }+\frac{r_{ijk}}{2}E_{j}E_{k}-q_{ij\lambda }E_{j}S_{\lambda }-\frac{g_{\lambda \nu i}}{2}S_{\lambda }S_{\nu }, \end{array}$$

having

(11) $$\begin{array}{c} \begin{array}{c} \frac{q_{ij\lambda }}{2}E_{j}E_{k}=\sum_{n=1}^{3}\frac{q_{nn\lambda }}{2}E_{n}E_{n} \end{array} \end{array}$$

In Equation (10), given the equivalence between the Voigt and traditional mechanical notation, an algebraic factor is not needed; this means

(12) $$\begin{array}{c} t_{\lambda \mu \nu }\equiv t_{ijklmn}\;,\;\;g_{\lambda \mu m}\equiv g_{ijklm}\;,\;\;q_{jk\lambda }\equiv q_{jklm}\;\;\forall \;\lambda ,\mu ,\nu \in \left[1,6\right] \end{array}$$

Equation (10) describes how the exchange of the coupling fields’ magnitudes is performed through the direct and converse piezoelectric effect, while the nonlinear contributions generated by the relatively high electric and deformation fields were considered. These conditions subject the material to mechanical and electrical stress, producing variations in all material parameters, as will be shown in the nonlinear effects section.

In this context, $t_{\lambda \mu \nu }$ is the contribution to the stress field due to strong deformations taking importance in the plastic operation regime. Furthermore, $t_{\lambda \mu \nu }$ relates the orthogonal deformations $S_{\mu }$ and $S_{\nu }$ that produce the change of the stress field with respect to the linear approximation. Analogously, $r_{ijk}$ is a correction term for the electric displacement vector as function of very high electric fields $E_{j}$ and $E_{k}$, so this tensor governs the dielectric polarization when $|E_{i}|$ is around $10^{9}$ V/m. $g_{\lambda \mu k}$ is responsible for the elasto-electric effect (in the literature, also known as nonlinear electrostriction and the electro-elastic effect), since its contribution to the stress field $g_{\lambda \mu k}\;S_{\mu }\;E_{k}$ provokes an augmentation of the effective elasticity constants, producing a stiffening of the material. In the same way, $q_{ij\lambda }E_{j}S_{\lambda }$ contributes to the electric permittivity due to the strains $S_{\lambda }$, and it is responsible for the change in the effective permittivity of a material subject to relatively high electric fields. Finally, The last quadratic terms of Equation (10) are a contribution to the stress and electric displacement field, modifying the value of the coupling piezoelectric coefficients $e_{k\lambda }$ and $e_{i\mu }^{T}$, respectively.

With this approach, the state equations presented remain balanced, while considering the nonlinear effects, and therefore, the physical behavior of the microscopic and macroscopic states of the physics system are predicted correctly.

## 3. Transformation Laws

To obtain the symmetry structure of any tensor, we need to know the transformation laws and the symmetry generators $a_{ij}$ of each crystal type (point group of symmetry). Taking into account the recommended notation for crystal classes and point groups by IUCr [39], $a_{ij}$ belongs to special orthonormal group SO(3), since it represents a generic 3D rotation. Furthermore, the transformation laws for the high-order tensors must meet the constraints of the positive energy and generate stable states for the system (e.g., the vanishing of the total torque about the origin), and their symmetry structure must only depend on the point group of the material. Then, to deduce the transformation laws for each high-order tensor, we start with the example of the calculus of the transformation law for the electrical permittivity of any material. The transformations laws for the electric displacement vector and electric field are

(13) $$\begin{array}{c} D_{i}^{{}^{\prime }}=a_{ij}D_{j}\;\;,\;\;\;E_{i}^{{}^{\prime }}=a_{ij}E_{j}, \end{array}$$

where the superscript ${}^{\prime }$ means a transformed magnitude. Now, knowing the law for the polarization of a material:

(14) $$\begin{array}{c} D_{i}=\epsilon_{ij}E_{j}, \end{array}$$

the target is to obtain an equivalent equation in terms of transformed magnitudes, so using Equation (13), we obtain

(15) $$\begin{array}{c} D_{i}^{{}^{\prime }}=\epsilon_{kj}a_{ik}{\left(a_{lj}\right)}^{-1}E_{l}^{{}^{\prime }}, \end{array}$$

where the transformation law for electrical permittivity is deduced from the symmetry condition, which means that, after transformation, the tensor form (structure) remains invariant:

(16) $$\begin{array}{c} \epsilon_{il}=\epsilon_{kj}a_{ik}{\left(a_{lj}\right)}^{-1} \end{array}$$

For the $q_{ij\lambda }$ high-order tensor, we need the transformation law for the deformation field:

(17) $$\begin{array}{c} S_{\mu }^{{}^{\prime }}=N_{\mu \nu }S_{\nu }, \end{array}$$

where $N_{\mu \nu }$ is a function of the symmetry generator $a_{ij}$ [38]. Then, using

(18) $$\begin{array}{c} D_{k}=-q_{kj\lambda }E_{j}S_{\lambda }, \end{array}$$

we obtain

(19) $$\begin{array}{c} D_{i}^{{}^{\prime }}=-a_{ik}q_{kj\lambda }{\left(a_{lj}\right)}^{-1}{\left(N_{\mu \lambda }\right)}^{-1}E_{j}S_{\lambda }, \end{array}$$

where the transformation law obtained for the $q_{ij\lambda }$ tensor is

(20) $$\begin{array}{c} q_{il\mu }^{{}^{\prime }}=q_{kj\lambda }a_{ik}{\left(a_{lj}\right)}^{-1}{\left(N_{\mu \lambda }\right)}^{-1} \end{array}$$

Through an analogous deduction, the transformation laws for the nonlinear tensors in the Equation (10) can be obtained, and they are shown below:

(21) $$\begin{array}{c} t_{\lambda \mu \nu }^{{}^{\prime }}=t_{\alpha \beta \gamma }\;\;M_{\lambda \alpha }N_{\beta \mu }^{-1}N_{\gamma \nu }^{-1}\;,\\ g_{\lambda \mu k}^{{}^{\prime }}=g_{\beta \nu m}\;\;M_{\lambda \beta }N_{\nu \mu }^{-1}a_{mk}^{-1}\;,\\ q_{jk\lambda }^{{}^{\prime }}=q_{lm\beta }\;\;M_{\lambda \beta }a_{lj}^{-1}a_{mk}^{-1}\;,\\ r_{ijk}^{{}^{\prime }}=r_{lmn}\;\;a_{il}a_{mj}^{-1}a_{nk}^{-1} \end{array}$$

At this point, the reader can notice that there are two ways to obtain the transformation laws, one per state equation in (10). Both ways have equivalent results knowing the properties of a symmetry generator $a_{ij}$ (belongs to the SO(3) group) and the *M* and *N* matrices [38]:

(22) $$\begin{array}{c} \begin{array}{c} {\left(a_{ij}\right)}^{-1}={\left(a_{ij}\right)}^{T}=a_{ji}\;,\;\;and\;\;{\left(N^{-1}\right)}_{ij}={\left(M^{T}\right)}_{ij} \end{array} \end{array}$$

## 4. Symmetry Structure for High-Order Tensors

The structure of the tensors can be calculated from Equation (21), the generator symmetry $a_{ij}$ for the specific crystal type, and a last mathematical constraint

(23) $$\begin{array}{c} -\frac{\partial^{2}D_{k}}{\partial S_{\lambda }\partial S_{\mu }}=g_{\lambda \mu k}=g_{\mu \lambda k},\\ -\frac{\partial^{2}T_{\lambda }}{\partial E_{k}\partial E_{j}}=q_{kj\lambda }=q_{jk\lambda },\\ -\frac{\partial^{2}D_{i}}{\partial E_{j}\partial E_{k}}=r_{ijk}=r_{ikj},\\ -\frac{\partial^{2}T_{\lambda }}{\partial S_{\mu }\partial S_{\nu }}=t_{\lambda \mu \nu }=t_{\lambda \nu \mu }, \end{array}$$

based on the mixed derivatives theorem. Then, selecting a transformation law from Equation (21) for the desired high-order tensor structure, a specific point group of symmetry (e.g., 6 mm), and applying Equation (23) in the transformation law selected, we obtain an undetermined algebraic linear system, which, after being solved, we obtain the structure of the tensor in terms of a few unique components, which represents the contribution of the specific tensor to the nonlinear behavior of the piezoelectric material. This procedure to obtain the symmetry structure of the high-order tensors was tested through obtaining the symmetry structure of known tensors for the thirty-two point groups; specifically, the elasticity constants, electrical permittivity, piezoelectric coupling coefficients, and $r_{ijk}$ tensor were reproduced; the last one is the only high-order tensor, whose complete symmetry structure has been published [41].

Table 1 presents a first approximation of the high-order tensors for two common piezoelectrics, PZT−5H and aluminum nitride (AlN), which belong to the 4 mm and 6 mm point groups, respectively. These results were obtained after reviewing the literature and noticing that, when an excitation signal provokes the appearance of the nonlinear effects [7,10,33], we suppose a variation around 2% for dependent variables with respect to the linear approximation.

Table 2 presents the symbols and particular numeration for the thirty-two point groups of symmetry; this numeration is used in the tables where the tensor structures are shown, and all high-order tensor components are introduced only by subscripts. The symmetry structures of tensors $q_{jk\lambda }$ and $g_{\lambda \nu i}$ are shown in Table 3 and Table 4, respectively; the first column contains the component of the high-order tensor and the following columns its corresponding value for a specific point group. The symmetry structure of $q_{jk\lambda }$ depends only on the Laue symmetry group. All types of crystalline materials have $q_{jk\lambda }$ and $t_{\lambda \mu \nu }$ different from zero in at least one component, and the $g_{\lambda \nu i}$ and $r_{ijk}$ tensors are null if the material does not exhibit linear piezoelectric behavior (this means they are centrosymmetric crystals), with the only exception of point group 432, where $g_{\lambda \nu i}$ is not zero and $r_{ijk}$ remains null. The symmetry structure of $t_{\lambda \mu \nu }$ for some point groups is shown in Appendix A in Table A1; the point groups not included are HI and RII; they need a separate complete analysis, and due to this, they are postponed for a future work.

## 5. Nonlinear Effects of Piezoelectric Materials

The nonlinear phenomena of the piezoelectric effect take importance when the material is subject to relatively high electric fields and strong deformations, and its consequences can be classified into two categories. First is the change of the mechanical and electrical properties such as the change of electrical permittivity, elasticity constants, and piezoelectric coupling coefficients. Second is the behavior variation of the physicalsystem response due to the modified material parameters, in particular the arising of the hysteresis behavior, changes in the electromechanical coupling factor, a shift of the resonance frequency, and the modification of the capacitance of the devices.

### 5.1. Variation of Mechanical and Electrical Properties

The change of the electrical permittivity in a piezoelectric material is produced by strong deformations or high temperatures [42] and can be induced by exciting the material with a relatively high electric field, the converse piezoelectric effect producing the deformations needed. This physical behavior can be observed from the nonlinear state equations, since the tensor $q_{jk\lambda }$ in the $D_{i}$ equation modifies the total polarization, and this can be integrated into a unique term with the electrical permittivity as follows:

(24) $$\begin{array}{c} \epsilon_{ij}^{eff}=\epsilon_{ij}-q_{ij\lambda }S_{\lambda }, \end{array}$$

where $\epsilon_{ij}^{eff}$ is the effective electrical permittivity.

The change of the elasticity constants is due to exposing the material to relatively high electric fields, which provokes a change in the interatomic electronic forces due to deformations, consequently causing a variation of the stiffness of the material. Furthermore, this phenomenon is included in the state equations through the modification of the total stress induced by the contribution of the deformations and can be formulated as effective elasticity constants as

(25) $$\begin{array}{c} C_{\lambda \mu }^{eff}=C_{\lambda \mu }+g_{\lambda \mu k}E_{k} \end{array}$$

Finally, due to the power balance of the nonlinear state equations, the variation of the piezoelectric coefficients is a consequence of the imbalance produced by the two last phenomena discussed, where the variation in the transduced power produced by the first nonlinear effect is compensated by the second, then the effective piezoelectric coupling coefficients are

(26) $$\begin{array}{c} e_{j\lambda }^{eff}=e_{j\lambda }-\frac{q_{kj\lambda }}{2}E_{k},\\ e_{i\mu }^{T-eff}=e_{i\mu }^{T}-\frac{g_{\lambda \mu i}}{2}S_{\lambda }, \end{array}$$

where $e_{j\lambda }^{eff}$ and $e_{i\mu }^{T-eff}$ must be used in the $T_{\lambda }$ and $D_{i}$ state equations, respectively.

### 5.2. Change Response of the PhysicalSystem

The literature shows how some piezoelectric devices that are subject to relatively high electric fields have a shift of their resonance frequency; this is produced by the variation of the electrical permittivity and the elasticity constants phenomenon explained before [20,43,44,45]. Generally, the resonance frequency of a piezoelectric resonator depends on its geometric length and the specific material, often calculated as

(27) $$\begin{array}{c} f_{r}=\frac{1}{2\lambda_{d}}\sqrt{\frac{C_{\lambda \mu }^{D-eff}}{\rho }}, \end{array}$$

where $\lambda_{d}$ is the wavelength of the device, ρ the density of the material, and

(28) $$\begin{array}{c} C_{\lambda \mu }^{D-eff}=C_{\lambda \mu }^{eff}+\frac{{\left(e_{k\lambda }\right)}^{2}}{\epsilon_{ij}}\;, \end{array}$$

s the effective elasticity constant (in some cases, it can be called the effective Young’s modulus), requiring all subscripts to match the main oscillation mode of the studied device. If we analyze the relative change of the resonance frequency (Equation (27)), we can obtain

(29) $$\begin{array}{c} \frac{df_{r}}{f_{r}}=\frac{dC_{\lambda \mu }^{D-eff}}{2C_{\lambda \mu }^{D-eff}}-\frac{d\rho }{2\rho }-\frac{d\lambda_{d}}{\lambda_{d}} \end{array}$$

From Equation (29), it can be noticed how the shift of the resonance frequency is a consequence of the changes of the effective elasticity constants, density, and wavelength of the device, where the last two terms are well known, so they can be neglected [31], because the piezoelectric materials are non-centrosymmetric crystals and the transverse/longitudinal dilatation does not provoke the measured order of magnitude for nonlinear effects.

The variation in the capacitance of the devices is explained through the change in the electrical permittivity phenomenon. Normally, the value of the capacitance of devices that have a dielectric as a piezoelectric material is

(30) $$\begin{array}{c} C^{eff}=\epsilon_{ii}^{eff}\frac{A}{t}, \end{array}$$

where $C^{eff}$ is the effective capacitance of the device, *A* is the electrodes’ contact area, *t* is the thickness of the piezoelectric, and $\epsilon_{ii}^{eff}$ is the effective electrical permittivity over the *i* axis. Performing an analogous relative variation analysis, then

(31) $$\begin{array}{c} \frac{dC^{eff}}{C^{eff}}=\frac{d\epsilon_{ii}^{eff}}{\epsilon_{ii}^{eff}}+\frac{dA}{A}-\frac{dt}{t}, \end{array}$$

where the last two terms can be neglected, inclusive of the nonlinear effects regime. This is due to the absolute displacement of particles because the order is 0.1Å (theoretical prediction) for the piezoelectric materials under these conditions; hence, dA and dt are not comparable with $d\epsilon_{ii}^{eff}$, since its variation is of the order of thousandths [20].

The calculus for the electromechanical coupling factor $k_{eff}^{2}$ is defined for resonant applications of piezoelectric materials and depends on the oscillation mode of the device, material properties, and specific device geometry. $k_{eff}^{2}$ is a measure of the exchange of power transduced between the mechanical and electrical fields, and for the most common devices, it has an expression of the form [38,46]

(32) $$\begin{array}{c} k_{eff}^{2}=\frac{{\left(e_{x5}^{eff}\right)}^{2}}{C_{44}^{eff}\epsilon_{xx}^{eff}}\;, \end{array}$$

for a device with Z-shear oscillation mode and a wave in the X-propagation axis. In Equation (32), the most significant variation, following the discussion above, comes from the effective elasticity constants [31]; hence, when a shift of the resonance frequency occurs, the electromechanical coupling factor increases its value, while the effective elasticity constants decrease. Therefore, for applications where power transduction is the main goal (e.g., energy harvester, microphones, etc.), it can be deduced using Equations (28) to (32) that a negative external electric field increases the performance of the device [13].

The appearance of the hysteresis behavior in the piezoelectric materials as a soft ferroelectric effect is a well-known phenomenon; this is produced by two main causes, the alignment of the dipoles in the unit cells of the material with respect to an external electric field and the change of the domain walls [47,48,49,50]. The change in the domain walls produces a spontaneous strain, inducing additional stress and polarization, and the alignment of the unit cells corresponds to the spontaneous polarization field having a contribution to the strain field. The correspondence between a cyclic electric field and the response of the polarization field and deformation field results in a hysteresis loop and butterfly loop, respectively [51]. The thermodynamic formulation presented only considers the spontaneous strain produced by high electric fields induced due to the inverse piezoelectric effect (last term of the $T_{\lambda }$ state equations), but it is only one of the theoretical treatments needed for a complete description of the hysteresis behavior.

In the context of all the experimental evidence exposed and discussed, the several nonlinear effects in piezoelectric materials take importance in different regimes. We describe the limit of the formulation presented as a function of the importance of the high-order tensors for their respective regime of operation, where the nonlinear electric contributions take precedence over the mechanical ones [52,53]. Taking as independent physical magnitudes the electrical and deformation field, if the material is subject to excitements of an order of magnitude under $10^{6}$ V/m and $10^{-6}$, respectively, the linear formulation would be enough. From there, the $g_{\lambda \nu i}$ and $q_{jk\lambda }$ tensors must be taken into account, where the $r_{ijk}$ domain makes electric contributions with electric fields above $10^{9}$ V/m, and $t_{\lambda \mu \nu }$ is only required starting from the plastic regime. Finally, the hysteresis behavior appears as a soft ferroelectric effect for some specific piezoelectric crystals with excitements of the order above $10^{6}$ V/m and $10^{-4}$ for the electric and deformation fields, respectively. It is necessary to bear in mind that, currently it is not clear what the starting point for the hysteresis behavior for any piezoelectric material is, since this effect belongs to the point group of the material or is induced by very high electric or deformation fields. The last discussion only applies to crystalline piezoelectric materials that are subject to nonlinear perturbative excitements.

## 6. Experimental Validation: Simulation

To validate the theoretical development performed in this work, we chose a reference that showed the nonlinear behavior of the piezoelectric devices under a relatively high electric field, since this is the simplest method to induce the nonlinear phenomena. The reference to reproduce is [28], where a solidly mounted resonator (SMR) was fabricated and characterized using AlN as a piezoelectric material; the fabrication details can be found in the reference. Measurements were performed with an Advantest R3767 S-Parameter analyzer, with the DC offset generated by a Keithley K327 and connected through a bias-T, and finally, the data acquisition was performed with the Picoprobe ECP18 GS-200 PP. Therefore, to implement the nonlinear state equations deduced, we can start neglecting the contributions of the $t_{\lambda \mu \nu }$ and $r_{ijk}$ tensors, since the operation regime and nonlinear behavior of the device are dominated by the linear description and the tensors $g_{\lambda \nu i}$ and $q_{jk\lambda }$ [52,53].

To include the nonlinear state equations within the simulations in an easy way, we chose the following formulation:

(33) $$\begin{array}{c} T_{\lambda }=C_{\lambda \mu }^{eff}S_{\mu }-e_{k\lambda }^{eff}E_{k},\\ D_{i}=\epsilon_{ij}^{eff}E_{j}+e_{i\mu }^{T-eff}S_{\mu }, \end{array}$$

where we used Equations (25) and (26), since this implementation included the power balance between the physics magnitudes of interest ($T_{\lambda }$ and $D_{i}$) within the FEM simulator. Generally, the FEM simulators allow us to set the electrical relative permittivity as an input parameter, so we rewrite $\epsilon_{ij}^{eff}$ as

(34) $$\begin{array}{c} \begin{array}{c} \epsilon_{ij}^{eff}=\epsilon_{0}\epsilon_{ij}^{r-eff}=\epsilon_{0}\left(\epsilon_{ij}^{r}-q_{ij\lambda }^{r}S_{\lambda }\right), \end{array} \end{array}$$

where $\epsilon_{0}$ is the vacuum electrical permittivity, $\epsilon_{ij}^{r}$ is the relative permittivity in the linear regime, $\epsilon_{ij}^{r-eff}$ is the effective relative permittivity, and the last term is defined as

(35) $$\begin{array}{c} \begin{array}{c} q_{ij\lambda }^{r}=\frac{q_{ij\lambda }}{\epsilon_{0}} \end{array} \end{array}$$

The SMR devices have the main oscillation mode, which confines the mechanical waves within the device; based on this, the algebraic tensor development of Equation (33) results in the only components of $g_{\lambda \nu i}$ and $q_{jk\lambda }$ that must be taken into account to be $g_{333}$, $q_{331}^{r}$, and $q_{333}^{r}$. The symmetry structure taken from Table 3 and Table 4 was the 6 mm one, since the piezoelectric material was AlN. The values obtained for the high-order tensors from the simulations were

(36) $$g_{333}=-80N/Vm,\;and\;q_{331}^{r}=q_{333}^{r}=-120$$

**Figure 1:**
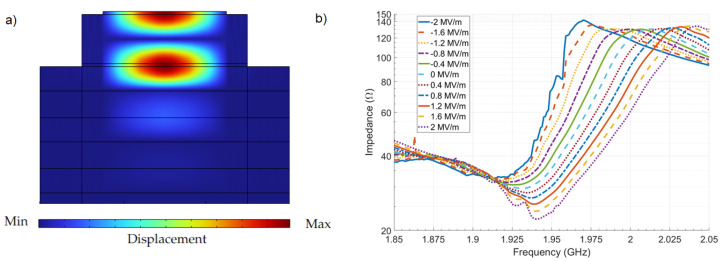
(**a**) Transversal cut of the simulated device that was fabricated in [28]; the scales for the axis are different to expose all the layers. (**b**) The impedance of the devices simulated for an external DC electric field in the range of −2 MV/m to 2 MV/m.

**Figure 2:**
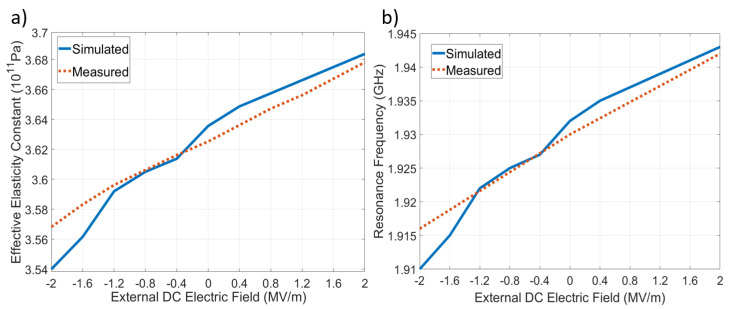
(**a**) Effective elasticity constants $C_{33}^{D-eff}$ and (**b**) resonance frequencies, obtained from the simulations and measurements of the device fabricated in [28], for an external DC electric field in the range of −2 MV/m to 2 MV/m.

**Figure 3:**
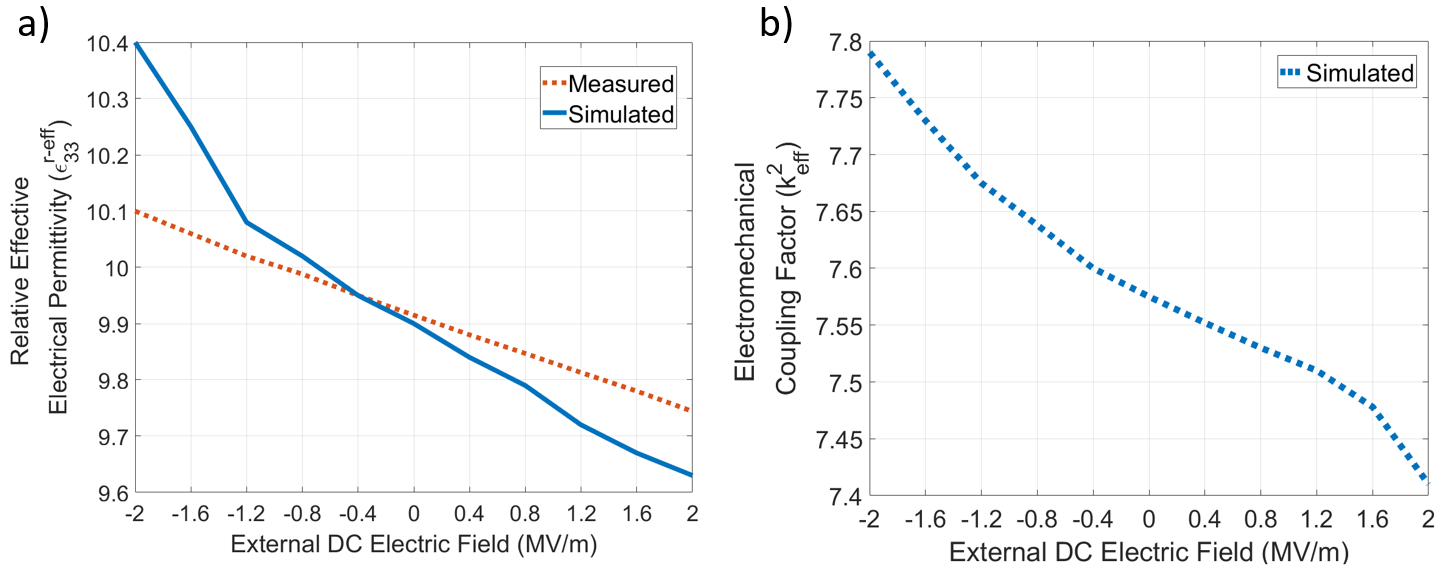
(**a**) Relative effective permittivity $\epsilon_{33}^{r-eff}$ obtained from the simulations and measurements of the device fabricated in [28] for an external DC electric field in the range of −2 MV/m to 2 MV/m. (**b**) Electromechanical coupling factor for the device simulated; this parameter was not reported by [28].

The SMR device was powered by an S-Parameter analyzer with a DC bias added with a bias-T through the signal probe of the RF microprobes. To reproduce the experimental setup, the simulated device was connected to an RF source with a DC voltage overlap, to calculate the whole interest frequency spectrum as a function of the DC bias. Figure 1a shows a transversal cut of the device simulated. In Figure 1b, the impedance of the device simulated for different DC biases is presented, and there, we can observe how the frequency response depends on the external DC electric field (EDEF), since it augments the stiffness of the material when positive voltages are applied, increasing the elasticity constants’ values, and consequently, the resonance frequency increases as well; this behavior’s prediction was performed by Equation (27). The effective elasticity constant $C_{33}^{D-eff}$ obtained from the measurements and simulations is shown in Figure 2a, where the maximum percent error obtained was 0.79%. The stiffening of the material was proportional to the EDEF due to the negative sign of $g_{333}$; consequently, the resonance frequency had the same dependency. This can be observed in Figure 2b, where the resonance frequencies measured and simulated, for several values of the EDEF, are presented; there, the maximum percent error obtained was 0.3%. The behavior obtained from the measurements and simulations for the relative effective permittivity is exposed in Figure 3a, where the linear inverse dependence between the EDEF and the permittivity can be observed, as predicted by Equation (24); the maximum percent error obtained was 2.9%. As can be expected, the slopes in Figure 2a and Figure 3a correspond to the values of $g_{333}$ and $q_{331}$, having the correct prediction for the trend behavior observed experimentally. Finally, the behavior of the electromechanical coupling factor is shown in Figure 3b, where the predictions of Equations (28) to (32) are corroborated, since the maximum value for $k_{eff}^{2}$ was obtained under negative voltages for the EDEF; this behavior was not reported by the experimental reference, but it was obtained from the simulations. In Table 5 is shown the average and maximum percent errors obtained from the simulations with respect to the measurements; there, the maximum percent error for the effective elasticity constants, effective relative permittivity, and resonance frequencies were 0.79%, 2.9%, and 0.3% respectively. These errors were caused by the difference between the physical material parameters and those used in the simulations; furthermore, the inaccuracy in the extremes of the values of the EDEF was due to the divergence problems that are present in the direct solver of the FEM software; this can be observed mainly in Figure 1b and Figure 3a. Nevertheless, in the scope of the simulations performed, the maximum percent error obtained for any material parameter or physicalsystem parameter was 1.1%; this shows the accuracy of the state equations presented to predict the main nonlinear phenomena of piezoelectric materials through a unified set of state equations, which can be included in FEM simulators easily.

## 7. Conclusions

In this work, we presented the nonlinear state equations for piezoelectric materials obtained from first-principles, conserving the power balance exchange between the dependent physical magnitudes $T_{\lambda }$ and $D_{i}$ and having a unified set of equations that predicts the behavior of the nonlinear phenomena. Furthermore, we showed how we obtained the transformation laws and the symmetry structures for the $r_{ijk}$, $g_{\lambda \mu k}$, and $q_{ij\lambda }$ tensors, while the calculation procedure was demonstrated with known tensor structures ($C_{\lambda \mu },e_{\lambda k}$, and $\epsilon_{ij}$). The physical connection and explanation for the nonlinear phenomena experimentally observed in the piezoelectric material were exposed, remarking on the excitement conditions that made each phenomenon appear, where, under an external DC electric field less of than $10^{9}V/m$, the nonlinear phenomena were dominated by the change in the relative effective permittivity and effective elasticity constants through the $g_{\lambda \mu k}$ and $q_{ij\lambda }$ tensors. The elastoelectric effect does not appear in non-piezoelectric materials ($g_{\lambda \mu k}$ is null), but the electrostrictive effect and nonlinear piezoelectric behavior remained within the material since qijλ was not zero, except for point group 432, where qijλ=0 and $g_{\lambda \mu k}$ was not null. A fast methodology for the implementation of the nonlinear state equations in the main FEM simulation software was exposed and demonstrated; this was carried out through the reproduction of an experimental reference, where the main nonlinearities of the piezoelectric effect were measured. The maximum percent errors obtained from the simulations were 0.79%, 2.9%, and 0.3% for the effective elasticity constants, relative effective permittivity, and resonance frequencies. This proved the effectiveness of the nonlinear stress–charge formulation presented, taking into account that the symmetry structure of each high-order tensor was shown (Table 3, Table 4 and Table A1). The design and simulation in the leading FEM simulators of nonlinear piezoelectric devices with a complete physical description are now possible.

## Figures and Tables

**Table 1:** Estimated order of magnitude of the high-order tensors for the nonlinear effects of AlN and PZT−5H (4 mm and 6 mm point groups respectively), using the stress–charge formulation presented.

Material Parameter	Symbol	Definition	Order	Units
Elasticity Constant	$C_{\lambda \mu }$	$\frac{\partial T_{\lambda }}{\partial S_{\mu }}$	$10^{11}$	N/m${}^{2}$
Relative Electrical Permittivity	$\epsilon_{ij}^{r}$	$\frac{\partial D_{i}}{\partial E_{j}}$	$10^{1}$	1
Electrical correction term of elasticity constants Strain correction term of piezoelectric coefficient	$g_{\lambda \mu k}$	$\frac{\partial^{2}T_{\lambda }}{\partial E_{k}\partial S_{\mu }}=\frac{\partial^{2}T_{\lambda }}{\partial S_{\mu }\partial E_{k}}$ $-\frac{\partial^{2}D_{k}}{\partial S_{\lambda }\partial S_{\mu }}$	$10^{1}-10^{3}$	C/m${}^{2}$
Electrical correction term of piezoelectric coefficient Strain correction term of electrical permittivity	$q_{ij\lambda }$	$-\frac{\partial^{2}D_{k}}{\partial S_{\lambda }\partial E_{j}}=-\frac{\partial^{2}D_{k}}{\partial E_{j}\partial S_{\lambda }}$ $\frac{\partial^{2}T_{\lambda }}{\partial E_{j}\partial E_{k}}$	$10^{-10}-10^{-8}$	N/Vm
Electrical correction term of permittivity	$r_{ijk}$	$\frac{\partial^{2}D_{i}}{\partial E_{j}\partial E_{k}}$	$10^{-20}-10^{-22}$	C/V${}^{2}$
Strain correction term of elasticity constants	$t_{\lambda \mu \nu }$	$\frac{\partial^{2}T_{\lambda }}{\partial S_{\mu }\partial S_{m}u}$	$10^{10}-10^{12}$	N/m${}^{2}$

**Table 2:** Numeration of point groups of crystal systems and Laue groups. The identification number corresponds to that used in Table 3, Table 4 and Table A1 to show the symmetry structure of the high-order tensors for each type of material.

Crystal System	Laue Group	Symbol	Id.	Symbol	Id.	Symbol	Id.
Triclinic	N	1	1	$\bar{1}$	2		
Monoclinic	M	2	3	m	4	2/m	5
Orthorhombic	O	222	6	mm2	7	mmm	8
Tetragonal	TII	4	9	$\bar{4}$	10	4/m	11
Tetragonal	TI	4 mm	13	$\bar{4}$2 m	14	4/mmm	15
Rhombohedral	RI	32	16	3 m	17	$\bar{3}$ m	18
Rhombohedral	RII	3	19	$\bar{3}$	20	422 ${}^{1}$	12
Hexagonal	HII	6	21	$\bar{6}$	22	6/m	23
Hexagonal	HI	622	24	6 mm	25	$\bar{6}$ m	26
Cubic	CII	23	28	m$\bar{3}$	29	6/mmm ${}^{2}$	27
Cubic	CI	432	30	4$\bar{3}$m	31	m$\bar{3}$m	32

^1^ Belongs to the TI Laue group. ^2^ Belongs to the HI Laue group.

**Table 3:** Symmetry structure of high-order tensor $q_{jk\lambda }$ for the nonlinear piezoelectric coupling effect.

Laue Group											
Comp.	N	M	O	TII	TI	RI	RII	HII	HI	CI	CII
111	111	111	111	111	111	111	111	111	111	111	111
121	121	0	0	121	0	0	121	121	0	0	0
131	131	131	0	0	0	0	131	0	0	0	0
221	221	221	221	221	221	221	221	221	221	221	221
231	231	0	0	0	0	231	231	0	0	0	0
331	331	331	331	331	331	331	331	331	331	331	331
112	112	112	112	221	221	221	221	221	221	331	331
122	122	0	0	−121	0	0	−121	−121	0	0	0
132	132	132	0	0	0	0	−131	0	0	0	0
222	222	222	222	111	111	111	111	111	111	111	111
232	232	0	0	0	0	−231	−231	0	0	0	0
332	332	332	332	331	331	331	331	331	331	221	221
113	113	113	113	113	113	113	113	113	113	221	221
123	123	0	0	0	0	0	0	0	0	0	0
133	133	133	0	0	0	0	0	0	0	0	0
223	223	223	223	113	113	113	113	113	113	331	331
233	233	0	0	0	0	0	0	0	0	0	0
333	333	333	333	333	333	333	333	333	333	111	111
114	114	0	0	0	0	114	114	0	0	0	0
124	124	124	0	0	0	0	124	0	0	0	0
134	134	0	0	134	0	0	134	134	0	0	0
224	224	0	0	0	0	−114	−114	0	0	0	0
234	234	234	234	234	234	234	234	234	234	234	234
334	334	0	0	0	0	0	0	0	0	0	0
115	115	115	0	0	0	0	−124	0	0	0	0
125	125	0	0	0	0	114	114	0	0	0	0
135	135	135	135	234	234	234	234	234	234	234	234
225	225	225	0	0	0	0	124	0	0	0	0
235	235	0	0	−134	0	0	−134	−134	0	0	0
335	335	335	0	0	0	0	0	0	0	0	0
116	116	0	0	116	0	0	−121	−121	0	0	0
126	126	126	126	126	126	A	A	A	A	234	234
136	136	0	0	0	0	231	231	0	0	0	0
226	226	0	0	−116	0	0	121	121	0	0	0
236	236	236	0	0	0	0	−131	0	0	0	0
336	336	0	0	0	0	0	0	0	0	0	0

A = (111−221)/2.

**Table 4:** Symmetry structure of the fifth-ranked tensor $g_{\lambda \mu k}$ for the nonlinear piezoelectric coupling effect. The point groups not shown are zero.

Point Group of Symmetry																				
Comp.	1	3	4	6	7	9	10	12	13	14	16	17	19	21	22	24	25	26	28,31	30
111	111	0	111	0	0	0	0	0	0	0	111	0	111	0	111	0	0	111	0	0
121	121	0	121	0	0	0	0	0	0	0	121	0	121	0	121	0	0	121	0	0
131	131	0	131	0	0	0	0	0	0	0	131	0	131	0	131	0	0	131	0	0
141	141	141	0	141	0	141	141	141	0	141	141	0	141	141	0	141	0	0	141	0
151	151	0	151	0	151	151	151	0	151	0	0	151	151	151	0	0	151	0	0	0
161	161	161	0	0	0	0	0	0	0	0	0	161	161	0	161	0	0	0	0	0
221	221	0	221	0	0	0	0	0	0	0	A	0	J	0	A	0	0	A	0	0
231	231	0	231	0	0	0	0	0	0	0	−131	0	−131	0	−131	0	0	−131	0	0
241	241	241	0	241	0	241	241	241	0	241	241	0	241	241	0	241	0	0	241	241
251	251	0	251	0	251	251	251	0	251	0	0	251	251	251	0	0	251	0	0	0
261	261	261	0	0	0	0	0	0	0	0	0	261	261	0	261	0	0	0	0	0
331	331	0	331	0	0	0	0	0	0	0	0	0	0	0	0	0	0	0	0	0
341	341	341	0	341	0	341	341	341	0	341	341	0	341	341	0	341	0	0	341	−241
351	351	0	351	0	351	351	351	0	351	0	0	342	351	351	0	0	351	0	0	0
361	361	361	0	0	0	0	0	0	0	0	0	361	361	0	361	0	0	0	0	0
441	441	0	441	0	0	0	0	0	0	0	441	0	441	0	441	0	0	441	0	0
451	451	451	0	0	0	0	0	0	0	0	0	451	451	0	451	0	0	0	0	0
461	461	0	461	0	461	461	461	0	461	0	0	E	E	E	0	0	E	0	0	0
551	551	0	551	0	0	0	0	0	0	0	−441	0	−441	0	−441	0	0	−441	0	0
561	561	561	0	561	0	561	561	561	0	561	B	0	B	B	0	B	0	0	561	0
661	661	0	661	0	0	0	0	0	0	0	121	0	121	0	121	0	0	121	0	0
112	112	112	0	0	0	0	0	0	0	0	0	F	F	0	F	0	0	0	0	0
122	122	122	0	0	0	0	0	0	0	0	0	G	G	0	G	0	0	0	0	0
132	132	132	0	0	0	0	0	0	0	0	0	361	361	0	361	0	0	0	0	0
142	142	0	142	0	142	251	−251	0	251	0	0	251	251	251	0	0	251	0	0	0
152	152	152	0	152	0	−241	241	−241	0	241	−241	0	−241	−241	0	−241	0	0	341	−241
162	162	0	162	0	0	0	0	0	0	0	C	0	C	0	C	0	0	C	0	0
222	222	222	0	0	0	0	0	0	0	0	0	H	H	0	H	0	0	0	0	0
232	232	232	0	0	0	0	0	0	0	0	0	−361	−361	0	−361	0	0	0	0	0
242	242	0	242	0	242	151	−151	0	151	0	0	151	151	151	0	0	151	0	0	0
252	252	252	0	252	0	−141	141	−141	0	141	−141	0	-141	−141	0	−141	0	0	141	0
262	262	0	262	0	0	0	0	0	0	0	D	0	-D	0	-D	0	0	-D	0	0
332	332	332	0	0	0	0	0	0	0	0	0	0	0	0	0	0	0	0	0	0
342	342	0	342	0	342	351	−351	0	351	0	0	342	351	351	0	0	351	0	0	0
352	352	352	0	352	0	−341	341	−341	0	341	−341	0	-341	−341	0	−341	0	0	241	241
362	362	0	362	0	0	0	0	0	0	0	−131	0	−131	0	−131	0	0	−131	0	0
442	442	442	0	0	0	0	0	0	0	0	0	−451	−451	0	−451	0	0	0	0	0
452	452	0	452	0	0	0	0	0	0	0	441	0	441	0	441	0	0	441	0	0
462	462	462	0	462	0	−561	561	−561	0	561	−B	0	−B	−B	0	−B	0	0	561	0
552	552	552	0	0	0	0	0	0	0	0	0	451	451	0	451	0	0	0	0	0
562	562	0	562	0	562	461	−461	0	461	0	0	E	E	E	0	0	E	0	0	0
662	662	662	0	0	0	0	0	0	0	0	0	G	G	0	G	0	0	0	0	0
113	113	0	113	0	113	113	113	0	113	0	0	113	113	113	0	0	113	0	0	0
123	123	0	123	0	123	123	0	0	123	0	0	123	123	123	0	0	123	0	0	0
133	133	0	133	0	133	133	133	0	133	0	0	133	133	133	0	0	133	0	0	0
143	143	143	0	0	0	0	0	0	0	0	0	143	143	0	143	0	0	0	0	0
153	153	0	153	0	0	0	0	0	0	0	153	0	153	0	153	0	0	153	0	0
163	163	163	0	163	0	163	163	163	0	163	0	0	0	0	0	0	0	0	241	241
223	223	0	223	0	223	113	−113	0	113	0	0	113	113	113	0	0	113	0	0	0
233	233	0	233	0	233	133	−133	0	133	0	0	133	133	133	0	0	133	0	0	0
243	243	243	0	0	0	0	0	0	0	0	0	−143	−143	0	−143	0	0	0	0	0
253	253	0	253	0	0	0	0	0	0	0	−153	0	−153	0	−153	0	0	−153	0	0
263	263	263	0	263	0	−163	163	−163	0	163	0	0	0	0	0	0	0	0	341	−241
333	333	0	333	0	333	333	0	0	333	0	0	333	333	333	0	0	333	0	0	0
343	343	343	0	0	0	0	0	0	0	0	0	0	0	0	0	0	0	0	0	0
353	353	0	353	0	0	0	0	0	0	0	0	0	0	0	0	0	0	0	0	0
363	363	363	0	363	0	0	363	0	0	363	0	0	0	0	0	0	0	0	141	0
443	443	0	443	0	443	443	443	0	443	0	0	443	443	443	0	0	443	0	0	0
453	453	453	0	453	0	0	453	0	0	453	0	0	0	0	0	0	0	0	561	0
463	463	0	463	0	0	0	0	0	0	0	−153	0	−153	0	−153	0	0	−153	0	0
553	553	0	553	0	553	443	−443	0	443	0	0	443	443	443	0	0	443	0	0	0
563	563	563	0	0	0	0	0	0	0	0	0	143	143	0	143	0	0	0	0	0
663	663	0	663	0	663	663	0	0	663	0	0	I	I	I	0	0	I	0	0	0

A = −111−121/2, B = −(141−241)/2, C = −111/2−121(3/2), D = (121−111)/2, E = (151−251)/2, F = 161/2 + 261(3/2), G = (161−261)/2, H = −261/2 161(3/2), I = (113−123)/2, J = −111 + 121/2.

**Table 5:** Percent errors obtained from the simulations with respect to the measurements for the effective elasticity constant, relative effective permittivity, and resonance frequency.

		Percent Error	
Quantity	Symbol	Average	Maximum
Effective Elasticity Constant	$C_{33}^{D-eff}$	0.28	0.79
Relative Effective Electrical Permittivity	$\epsilon_{33}^{r-eff}$	0.92	2.9
Resonance Frequency	$f_{r}$	0.15	0.3

## Data Availability

The data are contained within the article.

## Appendix A

The symmetry structure of $t_{\lambda \mu \nu }$ as function of the Laue group is shown in Table A1, where the expressions used are exposed in Table A2. The components of the tensor are listed as three numbers in a row, which correspond to their subscripts. The Laue groups HI and RII need a separate complete analysis; therefore, their structures are delayed for a future analysis.

**Table A1:** Symmetry structure of sixth-ranked tensor $t_{\lambda \mu \nu }$ for the nonlinear behavior of the stress field due to the strong deformation field. The expressions used are shown in Table A2.

Laue Group									
Comp.	N	M	O	TII	TI	RI	HII	CI	CII
111	111	111	111	111	111	111	111	111	111
121	121	121	121	121	121	121	121	121	121
131	131	131	131	131	131	131	131	121	131
141	141	0	0	0	0	141	0	0	0
151	151	151	0	0	0	0	0	0	0
161	161	0	0	161	0	0	161	0	0
221	221	221	221	221	221	221	221	221	221
231	231	231	231	231	231	231	231	231	231
241	241	0	0	0	0	241	0	0	0
251	251	251	0	0	0	0	0	0	0
261	261	0	0	261	0	0	261	0	0
331	331	331	331	331	331	331	331	221	331
341	341	0	0	0	0	341	0	0	0
351	351	351	0	0	0	0	0	0	0
361	361	0	0	361	0	0	361	0	0
441	441	441	441	441	441	441	441	441	441
451	451	0	0	451	0	0	451	0	0
461	461	461	0	0	0	0	0	0	0
551	551	551	551	551	551	551	551	551	551
561	561	0	0	0	0	561	0	0	0
661	661	661	661	661	661	661	661	551	661
112	112	112	112	221	221	A	A	221	331
122	122	122	122	121	121	B	B	121	131
132	132	132	132	231	231	231	231	231	231
142	142	0	0	0	0	C	0	0	0
152	152	152	0	0	0	0	0	0	0
162	162	0	0	−261	0	0	−161	0	0
222	222	222	222	111	111	D	D	111	111
232	232	232	232	131	131	131	131	121	121
242	242	0	0	0	0	E	0	0	0
252	252	252	0	0	0	0	0	0	0
262	262	0	0	−161	0	0	-261	0	0
332	332	332	332	331	331	331	331	221	221
342	342	0	0	0	0	−341	0	0	0
352	352	352	0	0	0	0	0	0	0
362	362	0	0	−361	0	0	−361	0	0
442	442	442	442	551	551	551	551	551	661
452	452	0	0	−451	0	0	−451	0	0
462	462	462	0	0	0	0	0	0	0
552	552	552	552	441	441	441	441	441	441
562	562	0	0	0	0	F	0	0	0
662	662	662	662	661	661	G	G	551	551
113	113	113	113	113	113	113	113	221	221
123	123	123	123	123	123	123	123	231	231
133	133	133	133	133	133	133	133	121	121
143	143	0	0	0	0	143	0	0	0
153	153	153	0	0	0	0	0	0	0
163	163	0	0	163	0	0	0	0	0
223	223	223	223	113	113	113	113	221	331
233	233	233	233	133	133	133	133	121	131
243	243	0	0	0	0	−143	0	0	0
253	253	253	0	0	0	0	0	0	0
263	263	0	0	−163	0	0	0	0	0
333	333	333	333	333	333	333	333	111	111
343	343	0	0	0	0	0	0	0	0
353	353	353	0	0	0	0	0	0	0
363	363	0	0	0	0	0	0	0	0
443	443	443	443	443	443	443	443	551	551
453	453	0	0	0	0	0	0	0	0
463	463	463	0	0	0	0	0	0	0
553	553	553	553	443	443	443	443	551	661
563	563	0	0	0	0	143	0	0	0
663	663	663	663	663	663	H	H	441	441
114	114	0	0	0	0	114	0	0	0
124	124	0	0	0	0	124	0	0	0
134	134	0	0	0	0	134	0	0	0
144	144	144	144	144	144	144	144	144	144
154	154	0	0	0	0	0	154	0	0
164	164	164	0	0	0	0	0	0	0
224	224	0	0	0	0	I	0	0	0
234	234	0	0	0	0	−134	0	0	0
244	244	244	244	244	244	244	244	244	244
254	254	0	0	254	0	0	254	0	0
264	264	264	0	0	0	0	0	0	0
334	334	0	0	0	0	0	0	0	0
344	344	344	344	344	344	344	344	244	344
354	354	0	0	354	0	0	354	0	0
364	364	364	0	0	0	0	0	0	0
444	444	0	0	0	0	444	0	0	0
454	454	454	0	0	0	0	0	0	0
464	464	0	0	464	0	0	U	0	0
554	554	0	0	0	0	−444	0	0	0
564	564	564	564	564	564	J	J	564	564
664	664	0	0	0	0	124	0	0	0
115	115	115	0	0	0	0	0	0	0
125	125	125	0	0	0	0	0	0	0
135	135	135	0	0	0	0	0	0	0
145	145	0	0	−254	0	0	−254	0	0
155	155	155	155	244	244	244	244	244	344
165	165	0	0	0	0	K	0	0	0
225	225	225	0	0	0	0	0	0	0
235	235	235	0	0	0	0	0	0	0
245	245	0	0	0	0	0	−154	0	0
255	255	255	255	144	144	144	144	144	144
265	265	0	0	0	0	L	0	0	0
335	335	335	0	0	0	0	0	0	0
345	345	0	0	−354	0	0	−354	0	0
355	355	355	355	344	344	344	344	244	244
365	365	0	0	0	0	134	0	0	0
445	445	445	0	0	0	0	0	0	0
455	455	0	0	0	0	−444	0	0	0
465	465	465	465	564	564	J	J	564	564
555	555	555	0	0	0	0	0	0	0
565	565	0	0	−464	0	0	−U	0	0
665	665	665	0	0	0	0	0	0	0
116	116	0	0	116	0	0	Y	0	0
126	126	0	0	0	0	0	X	0	0
136	136	0	0	136	0	0	−361	0	0
146	146	146	0	0	0	0	0	0	0
156	156	0	0	0	0	−E/2 + 241/2	0	0	0
166	166	166	166	166	166	D/2−111/ 4−221/4	D/2−111/ 4−221/4	244	244
226	226	0	0	−116	0	0	−3Y−4 × 261	0	0
236	236	0	0	−136	0	0	361	0	0
246	246	246	0	0	0	0	0	0	0
256	256	0	0	0	0	−C/2 + 141/2	0	0	0
266	266	266	266	166	166	N/2	N/2	244	344
336	336	0	0	0	0	0	0	0	0
346	346	346	0	0	0	0	0	0	0
356	356	0	0	0	0	341	0	0	0
366	366	366	366	366	366	O	O	144	144
446	446	0	0	446	0	0	451	0	0
456	456	456	456	456	456	P	P	564	564
466	466	0	0	0	0	M/2	0	0	0
556	556	0	0	−446	0	0	−451	0	0
566	566	566	0	0	0	0	0	0	0
666	666	0	0	0	0	0	X	0	0

**Table A2:** Abbreviated expressions used in Table A1 to show the $t_{\lambda \mu \nu }$ components.

Expression	Equivalence	Expression	Equivalence	Expression	Equivalence
A	121/2−111/4 + 3/4 × 221 + 661	J	(244−144)/2	R	−151/2−251/2−461
B	111/4 + 121/2 + 221/4−661	K	(114 + 3 × 124)/2	S	251/2−151/2
C	561−241/2−141/2	L	(114−124)/2	T	141/2−241/2
D	3/4 × 111 + 121/2−221/4 + 661	M	241/2−141/2 + 561	U	(154−254)/2
E	−141/2−241/2−561	N	(3 × 111)/4−121/2−221/4−661	V	−164/2−(3 × 264)/2
F	(141−241)/2	O	(131−231)/2	W	264/2−164/2
G	(111−2 × 121 + 221)/4	P	(551−441)/2	X	(261−161)/2
H	(113−123)/2	Q	461−251/2−151/2	Y	−(161 + 3 × 261)/2
I	−(114 + 2 × 124)				
